# 2-DE Mapping of the Blue Mussel Gill Proteome: The Usual Suspects Revisited

**DOI:** 10.3390/proteomes3010003

**Published:** 2015-01-12

**Authors:** Béatrice Rocher, Florence Bultelle, Philippe Chan, Frank Le Foll, Julie Letendre, Tiphaine Monsinjon, Stéphanie Olivier, Romain Péden, Agnès Poret, David Vaudry, Thomas Knigge

**Affiliations:** 1 Laboratory of Ecotoxicology—Aquatic Environments, UMR I-02 SEBIO, Le Havre University, 76063 Le Havre, France; E-Mails: florence.bultelle@univ-lehavre.fr (F.B.); frank.lefoll@univ-lehavre.fr (F.L.F.); julie.letendre@hotmail.com (J.L.); tiphaine.monsinjon@univ-lehavre.fr (T.M.); stephanie.olivier@univ-lehavre.fr (S.O.); romain.peden@univ-lehavre.fr (R.P.); agnes.poret@univ-lehavre.fr (A.P.); thomas.knigge@univ-lehavre.fr (T.K.); 2 PISSARO Proteomic Platform, Institute for Research and Innovation in Biomedicine, University of Rouen, 76821 Mont-Saint-Aignan, France; E-Mail: philippe.chan@univ-rouen.fr; 3 Neuronal and Neuroendocrine Differentiation and Communication (DC2N), Inserm U982, University of Rouen, 76821 Mont-Saint-Aignan, France; E-Mail: david.vaudry@univ-rouen.fr

**Keywords:** 2-DE, mass spectrometry, proteoforms, post-translational modifications, bivalve mollusc, environmental stress, ecotoxicology

## Abstract

The Blue Mussel (*Mytilus edulis*, L. 1758) is an ecologically important and commercially relevant bivalve. Because of its ability to bioconcentrate xenobiotics, it is also a widespread sentinel species for environmental pollution, which has been used in ecotoxicological studies for biomarker assessment. Consequently, numerous proteomics studies have been carried out in various research contexts using mussels of the genus *Mytilus*, which intended to improve our understanding of complex physiological processes related to reproduction, adaptation to physical stressors or shell formation and for biomarker discovery. Differential-display 2-DE proteomics relies on an extensive knowledge of the proteome with as many proteoforms identified as possible. To this end, extensive characterization of proteins was performed in order to increase our knowledge of the *Mytilus* gill proteome. On average, 700 spots were detected on 2-DE gels by colloidal blue staining, of which 122 different, non-redundant proteins comprising 203 proteoforms could be identified by tandem mass spectrometry. These proteins could be attributed to four major categories: (*i*) “metabolism”, including antioxidant defence and degradation of xenobiotics; (*ii*) “genetic information processing”, comprising transcription and translation as well as folding, sorting, repair and degradation; (*iii*) “cellular processes”, such as cell motility, transport and catabolism; (*iv*) “environmental information processing”, including signal transduction and signalling molecules and interaction. The role of cytoskeleton proteins, energetic metabolism, chaperones/stress proteins, protein trafficking and the proteasome are discussed in the light of the exigencies of the intertidal environment, leading to an enhanced stress response, as well as the structural and physiological particularities of the bivalve gill tissue.

## 1. Introduction

Molluscs belong to one of most diverse animal phyla and are a major component of marine ecological communities, comprising about a quarter of the known marine species. Within this clade, filter-feeding bivalves represent one of the dominating benthic life forms with fundamental importance for the marine ecosystem. Bivalves also provide a valuable food source to humans with numerous important fishery and aquaculture species. Consequently, several bivalve species, such as scallops, oysters and mussels are amongst the most studied marine organisms. In spite of this prominence, the mollusc and, specifically, bivalve genome remains poorly studied [1]. This is even more surprising in view of the fact that various mollusc species serve as models for studying neurobiology, biomineralisation, the adaptation to natural stresses of the coastal environments, ocean acidification and climate change as well as marine pollution [2].

Members of the genus *Mytilus* are used worldwide as sentinels in the biomonitoring of marine pollution (*i.e.*, Mussel Watch Project, Ifremer, France: “Réseau d'Observation de la Contamination Chimique”), on the one hand for their pervasive geographical distribution and, on the other hand, because they are highly tolerant to xenobiotics, which they bioaccumulate into considerable concentrations. As mussels are sessile, attached to a rocky substrate by their byssus threads, they also allow for spatial localisation of marine pollution, reflecting changes in the contamination of the environment from which they originate. These properties make them useful bioindicators of chemical pollution as well as useful models in ecotoxicology [3,4,5,6].

Mussels are naturally exposed to fluctuations of numerous abiotic factors, such as oxygen availability, temperature and salinity changes, which follow the rhythms in the intertidal zones [7]. Significant changes of body temperature of more than 20 °C occur, both when exposed to warm air and/or heated by solar radiation as well as when immersed into cold water afterwards [8,9]. Emersion and reimmersion are also accompanied by changes in oxygenation and metabolic activity, leading to oxidative stress [10,11]. Furthermore, blue mussels can cope with wide ranges of salinity allowing them to occupy brackish habitats of the estuaries. Hence, these animals provide an excellent model to study the stress response in dynamically changing environments as well as questions of adaptation to a life in the extremes [12]. The variability of stressful conditions is likely to stimulate quantitative changes in many different proteins at any one time [13]. In view of this complexity of interdepending mechanisms of the stress response, a systems biology approach is likely to provide a more comprehensive insight into the underlying molecular regulatory networks. Proteomics may thus capture the complexity of these stress responses better than a battery of individual assays.

Since *Mytilus* ssp. is cosmopolitan, different species can be found on the shores around the world and their thermal adaptation and oxidative stress response is likely to differ between species according to their distribution range [9,14,15]. Climate change is assumed to shift the range limits of the geographical distribution of *Mytilus* species with the thermal adaption of the congeners mostly determining their ability to invade new regions or to occupy different, *i.e.*, subtidal and intertidal, habitats [9,16,17]. Also, where the biogeographic ranges overlap, *Mytilus* congeners may hybridise, with the hybrids possibly having specific ecophysiological properties. Extensive hybridisation occurs between *M. edulis* and *M. galloprovincialis* along the coasts of Western Europe as well as between *M. trossulus* and *M. galloprovincialis* in the Baltic sea, along the west coast of North America, in Japan and adjoining coastal areas [16,18,19]. As *M. galloprovincialis* is often outgrowing the native species, it is also considered a problematic invasive species [16,20]. Proteomics has been employed to investigate *Mytilus* ssp. and to distinguish hybridisation forms [21,22,23]. Hybridisation also involves mechanisms of doubly uniparental mtDNA inheritance, a particularity of certain bivalve orders, including Mytiloida [19,24]. This phenomenon represents another aspect of *Mytilus* biology to which proteomics has been applied [24,25].

Although *Mytilus* spp. is considered a key model organism for molluscan biology [12] and is deemed to be an upcoming model organism [26], proteomics studies using *Mytilidae* are still hampered by limited knowledge on mussel genes as well as their expression in natural or polluted conditions. Notwithstanding the numerous studies that have focused on proteomic changes in mussels within the contexts outlined above, relatively few protein identifications have been accomplished in regard to the thousands of proteins present in a mussel (reviewed in [14,23,27,28]). In the present study, we intended to expand the description of the mussel gill proteome using gel-based proteomics. Although proteome coverage may be extended by mass-spectrometry based shotgun proteomics, 2-DE remains a valuable top-down proteomics approach [29]. On all accounts, it persists as the most commonly used technique in environmental proteomics. In addition, it may be particularly suited for the identification of both PTMs and protein isoforms as a result of environmental and/or pollution stress, which, however, have been scarcely investigated to date.

In filter-feeding bivalves, the gills are one of the major sites of interaction with the environment [30]. Their ctenidiae consist of lamellae, made up of ciliated filaments that create water currents in the pallial cavity, which are used both for breathing and feeding. Besides the gas exchange over the gill epithelia, the suspended food particles are retained in the gill mucus, sorted and transported by the cilia to the mouth [17,31]. Blue mussels can filter up to 5 L of seawater per hour over a large surface area. Consequently, the bivalve gills are one of the primary organs to be exposed to abiotic stressors such as thermal stress and desiccation as well as oxidative stress. Also, they are one of the major organs to be exposed to pollutants. These properties make them particularly interesting for studying proteomic alterations in relation to environmental and anthropogenic stressors. The findings of the present study will be discussed in light of the structural and functional characteristics of the bivalve gills.

## 2. Experimental Section

### 2.1. Chemicals

Reagents were purchased from GE Healthcare (Vélizy-Villacoublay, France) except acetonitrile (ACN) and trypsin, which were obtained from Thermo Fisher Scientific (Villebon-sur-Yvette, France) and Promega (Charbonnières, France), respectively. All chemicals used were of the highest grade available.

### 2.2. Animals and Sample Preparation

Adult blue mussels *Mytilus edulis* (4–5 cm shell length) were collected on the seashore of Yport, France (49°44' N; 0°18' E). It was approved by the ethics committee for animal experimentation of Normandy University that the use of bivalves in this study conforms to the European Directive 2010/63/EU concerning the care and use of laboratory animals under the French law on ethics of animal experimentation. The mussels were transported to the laboratory in aerated seawater from the sampling site at the pre-existing temperature. Upon arrival, the mussels were dissected immediately and gills were homogenized mechanically using an electric potter, in 50 mM Tris buffer, pH 7.5, containing 9 M urea, 2% (w:v) CHAPS, 2% (v:v) 2-β mercaptoethanol, 8 mM PMSF, 0.8% (v:v) pharmalytes pH 3–10 and protease inhibitor (16 µg·mL^−1^ aprotinin). The homogenates were stored on ice and sonicated for 30 s twice (Ultrasonic processor, Fischer-Bioblock, Aubagne, France). Cellular debris was removed by centrifugation at 9000× *g* for 15 min at 4 °C and the supernatants stored at −80 °C until further analysis. Protein concentrations were determined according to the method of Bradford [32] with bovine serum albumin as a standard.

### 2.3. Gel Analysis

For preparative gels, the homogenates were adjusted to 750 µg of total protein with rehydratation buffer containing 9 M urea, 2% (w:v) CHAPS, 65 mM dithioerythreitol, immobilised pH gradient (IPG)-buffer and loaded on 18 cm non-linear wide-range Immobiline Drystrips (pH 3–10, NL/18 cm; GE Healthcare), for overnight passive rehydration. Isoelectric focussing was carried out at 20 °C using a horizontal Multiphor electrophoresis apparatus (Amersham Pharmacia Biotech) according to the manufacturer’s recommendations. Subsequently, IPG strips were incubated in 15 mM dithioerythreitol in equilibration buffer (50 mM Tris buffer, pH 6.8, containing 6 M urea, 4% SDS, 25% glycerol) for 12 min followed by 120 mM iodoacetamide and bromophenol blue in equilibration buffer for 5 min. Second dimension was carried out with 12% SDS-PAGE gels (20 cm × 20 cm × 1.5 cm) at 10 °C, using a Protean Plus Dodeca cell (BioRad, Marnes-la-Coquette, France) at 600 mA for 15 min, followed by 1 A for 15 min and 200 V for 6 h, until the dye front reached the bottom of the gel. The protein spots were visualized by colloidal blue staining and scanned to TIFF images using an Image Scanner (Amersham Pharmacia Biotech). Image analyses were performed using Image Master 2-DE analysis software (Amersham Pharmacia Biotech). In total, five series of 12 gels each were processed and aligned using landmark protein spots. Relative abundance of individual spots was determined against the total spot volume, *i.e.*, the sum of all spots detected on the gel, and the standard deviation in relative abundance was calculated over all gels of the five series. To determine the relative dispersion within spot intensities, the coefficient of quartile variation (cqv) was applied as a non-parametric measure of variation: cqv = [(Q3 − Q1)/(Q3 + Q1)] × 100, where Q1 and Q3 are first and third population quartiles and Q3−Q1 is the interquartile range [33].

### 2.4. Mass Spectrometry and Protein Identification

In total, 313 different spots were selected and excised manually. Spots were required to appear repeatedly in 95% of the gels (within a series of a single run and amongst the different runs), to be well defined and separated as to be picked as individual spots and to provide sufficient material for subsequent protein identification. Duplicate identifications of identical spot positions deriving from different gels were carried out for the majority of spots. Proteins spots were digested by trypsin as follows: spots were washed 3 times for 15 min in water, twice in destain solution containing 100 mM ammonium bicarbonate and 50% (v:v) ACN and once in ACN. Proteins were then dried in a speed-vac concentrator (Thermo Fisher Scientific) for 5 min, followed by in-gel overnight digestion in 30 µL of a digestion buffer containing 50 mM ammonium bicarbonate and 6 ng/µL sequencing grade modified porcine trypsin. The digestion mixture was extracted with 50% (v:v) ACN and 5% (v:v) formic acid and then dried in a speed-vac. Peptide extracts were then resuspended in 12 μL of 3% (v:v) ACN/0.1% (v:v) formic acid and then analysed with a nano-LC1200 system coupled to a 6340 Ion Trap mass spectrometer equipped with a nanospray source and an HPLC-chip cube interface (Agilent Technologies, Les Ulis, France) as described previously [34]. The five most intensive peaks were subjected to MS/MS after two spectra with a dynamic exclusion time of one minute. The peptide fingerprints were analysed using MASCOT software with the following specifications: enzyme specificity, trypsin; one missed cleavage permitted; carbamidomethylation as fixed modification, methionine oxidation as variable modification; peptide charge, 2+ and 3+; mass accuracy of 1.6 Da for the parent ions (MS) and 0.6 Da for the fragment ions (MS/MS); ESI-TRAP as instrument; SwissProt and NCBInr as databases; “other metazoan” as taxonomy (SwissProt: 540261 sequences; 191876607 residues and NCBInr: 26.236.801 sequences; 9.088.244.489 residues, respectively). To improve identification, a second search was conducted with the same specifications against EMBL invertebrate EST database (95.448.618 sequences; 18.505.270.330 residues). The amino-acid sequences obtained were used to carry out a MS BLAST-PROT search in NCBI standard Protein Blast to identify proteins by sequence similarity against the available sequence databases. Identified proteins were further confirmed by the number of peptide matches, the degree of protein coverage and the accordance of actual and expected molecular mass (Mr) and isoelectric point (pI). Protein function was analysed with the KEGG Pathway database (http://www.genome.jp/kegg/ pathway.html).

## 3. Results and Discussion

**Figure 1:**
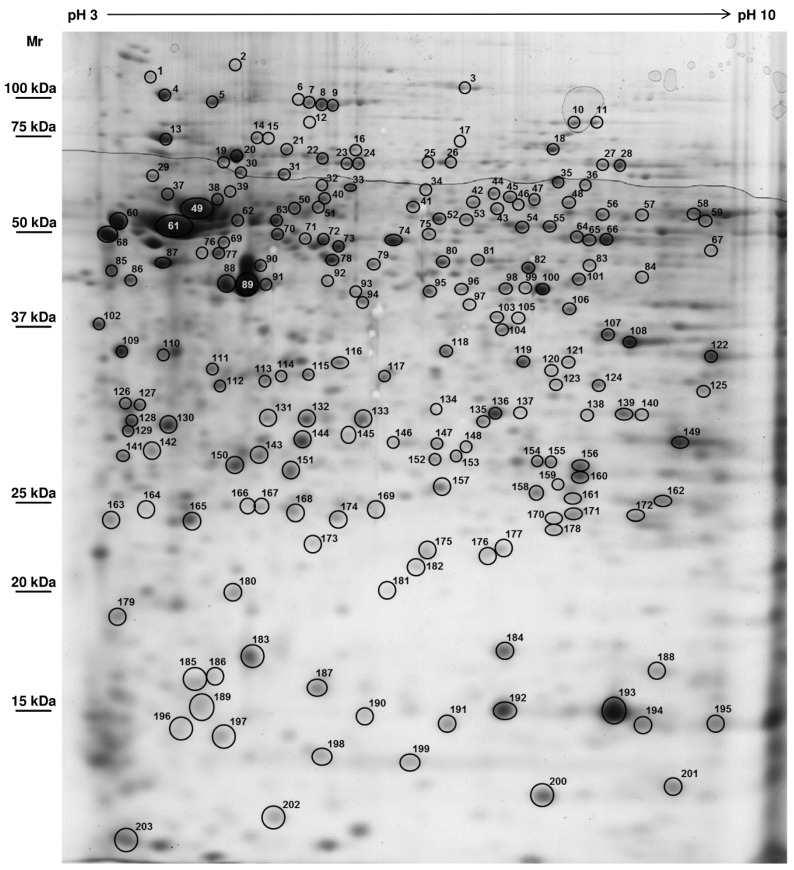
Representative *Mytilus edulis* gill proteome (750 µg total protein, non-linear pH 3–10, 12% SDS-PAGE, colloidal blue stain; [11]. Spot numbers correspond to identified proteoforms listed in Table 1. The isoelectric point is indicated on the horizontal axis and the molecular mass (Mr in kDa) on the vertical axis.

**Figure 2:**
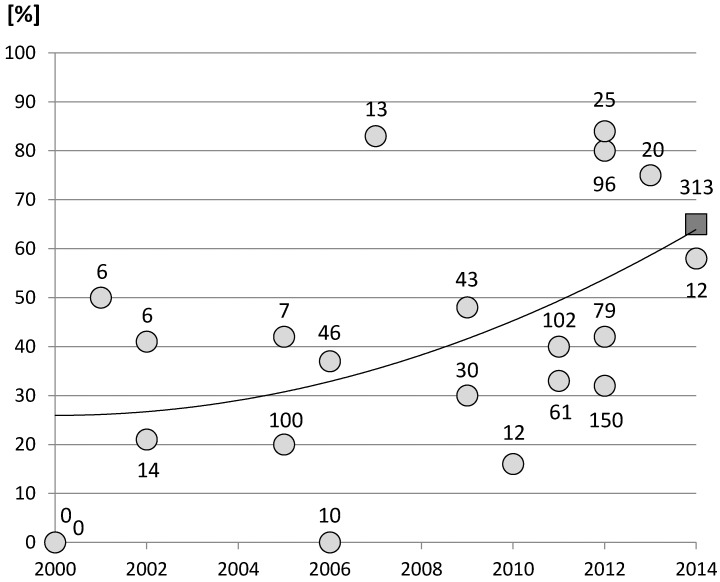
Number of spots identified by proteomics studies in the years 2000–2014 using the *Mytilus* complex (*i.e.*, *Mytilus edulis*, *Mytilus galloprovincialis*, *Mytilus trossulus* and hybrids thereof) [4,11,21,35,37,39,41,42,51,56,57,58,59,60,61,62,63,64,65]: the numerical data represent the number of proteins submitted to identification for each study. The 2nd order polynomial function illustrates the trend to an increased percentage of spots identified for *Mytilus* species. The dark grey square depicts the percentage of identification of the present study (65%) for which a total of 313 different spots were submitted for identification by nanoLC-MS/MS.

The *Mytilus* gill proteome was assessed using the same methodology as previously described [4,11], which allowed for cross-validation of identified proteins. However, protein extraction has been standardised and improved so as to obtain highly reproducible gels. Figure 1 shows a representative 2-DE gel from *M. edulis* gills in the broad pH range of 3–10 with around 700 spots visualized [11]. The ensemble of spots is distributed throughout the gel with well-defined spots. The global pattern is in general agreement with the profiles represented in the literature for the genus *Mytilus* since the first study of Shepard *et al.* [35]. Numerous other studies have analysed the mussel proteome thereafter, mostly in an ecophysiological [14,21,25,36] or ecotoxicological context [11,13,37,38,39,40,41,42]. The total number of proteins identified has been increasing continuously across these studies with percentage of identification now mostly exceeding 50% of spots obtained with gel-based proteomics (Figure 2). For this study, more than 300 different spots were submitted to identification of which 268 spots could be identified. However, about one quarter of the identifications resulted in ambiguous identifications relying on one single peptide only with multiple matches to different proteins. Protein identifications by a single peptide have been retained only in some exceptional cases, where one distinct protein emerged by a clearly higher Mascot score, thus permitting a distinction from the other identifications. The identification for these spots (no. 16, 31, 176 and 184) must be considered only as tentative (Table 1). Eventually, the identification of 203 different proteoforms was considered reliable (Table 1). These comprised 150 different subunits or isoforms, respectively, of a total of 122 different proteins. To the best of our knowledge, this study represents the most comprehensive proteome coverage for the genus *Mytilus*. The percentage of identification of 65% of the proteins is in line with the general development of protein identification for *Mytilus* species (Figure 2), as sequence information on molluscs in general has been improving continuously and expressed sequence tags (ESTs) of *Mytilidae* now amount to more than 70,000 sequences. Most of the identifications were thus either from mollusc species (59%), from bivalves (54%) or directly from *Mytilidae* (29%). The remaining protein spots were either unreliable identifications (21%) or proteins for which no match at all could be obtained (14%). The fact that more than one third of the selected protein spots could not be identified reflects that genomic information for this non-model organism, which has not yet been fully sequenced, is still lacking.

In terms of abundance the 203 identified proteoforms accounted for 40% of the total protein on the gels, with of actin and tubulin representing >4% and >4.5%, respectively; cytoskeleton proteins as a whole amounted to ca. 13% (Table 1). The majority of proteins identified were below 1% of the total protein, except for tubulin α-1 chain (spot no. 49), cytoplasmic β-actin (spot no. 89) and tubulin β chain (spot no. 61), the latter, with >3%, being the most abundant protein (Table 1).

Nearly 70% of the proteins represented a reasonable cqv below 20%, but only about 10% (21 proteins) were highly reproducible with a cqv below 10% (Table 2). On the other hand, almost one third of the proteins (*i.e.*, 62) showed a cqv beyond 20%, indicating that these proteins are highly variable in their abundance, which limits their value for quantitative studies.

Several proteoforms identified with a relatively high confidence exhibited nonetheless important deviations from the expected Mr and pI. Considerably lower Mr than predicted suggest the presence of truncated proteoforms, which might be the result of protein degradation, as for instance Grp94, which is found predominantly in spot no. 4 (Mr 95.115 Da) but appears also in spot no. 186 (Mr 16.505 Da). It is, however, difficult to interpret such protein degradation as either being due to possible degradation during extraction or to cellular processes prior to protein extraction. The spots no. 150 and 151, identified as actin, give a pertinent example for this problem of interpretation. The corresponding spectra resulted in an unambiguous identification, matching exclusively with actin, although the spots revealed much lower Mr than the major actin spot no. 89. The fact that the tryptic peptides covered the entire span of the protein, suggests that these spots may not represent a degraded form of actin. Alternatively, they may either represent fragments of actin or proteins that share common sequences, *i.e.*, actin-like proteins.

Shifting of proteins relative to their expected Mr and pI may also be due to amino acid sequence differences and/or post-translational modifications (PTMs) that may alter protein migration in both, horizontal and vertical directions. In fact, a considerable number of spots resulted in identical protein identifications (see also following sections), although they were recovered from more or less different positions on the gel. It is conceivable that this dispersion of spots relates to a variable degree and kind of PTMs for the respective proteins. In the case of one of the most frequently encountered PTMs, *i.e.*, phosphorylation, a monoisotopic mass difference of +79.966 Da is added, which does not affect Mr significantly, but will reduce the pI. Thus, phosphorylated proteins may appear as a characteristic line of horizontal spots depending on their degree of phosphorylation (Figure 1). This may be the case for spots no. 6, 7, 8, 9: major vault protein, spots no. 10 and 11: aconitase, for spots no. 23 and 24: hsp70 and for spots no. 27 and 28: phosphoenolpyruvate kinase, all of which are known to be phosphorylated [43,44,45,46]. These findings emphasise the potential of studying PTMs in differential-display 2-DE proteomics. Even the usual “*déjà vu*” proteins [47], such as actin, may contain important information concerning the PTMs. Environmental stress conditions are likely to induce different PTMs to these well-known proteins, amongst them notably the above mentioned phosphorylations [48,49]. Accordingly, ubiquitination and carbonylation/glutathionylation have been used in targeted redox proteomics [28,50,51,52,53]. Alternative to the quest for new marker proteins, which are not likely to be found amongst the prevalent canonical proteins that are typically revealed by conventional gel-based proteomics, PTMs could be highly informative in the biomonitoring of environmental changes and therefore deserve to be given more attention [54,55]. Furthermore, as long as limited genomic information still hampers sequence-homology searches, the analysis of PTMs of highly conserved proteins, which can be identified unambiguously, is a promising option to evaluate an organism’s health or physiological state. This would constitute a particular strength of proteomics, since the focus would be not on the induction of genes or the quantity of a given protein, but on protein function and its regulation as well as modification.

**Table 1:** Protein spots identified by tandem mass spectrometry in *Mytilus edulis* gills. Proteins were designated according to NCBI entries and classified with KEGG Pathway database. Spot number code for identification corresponds to spot numbers as depicted in Figure 1. Mr: molecular mass; pI: isoelectric point; obs.: observed; calc.: calculated; seq.: number of matched peptide sequences; cov.: sequence coverage in %; rel. Ab.: relative abundance in ‰ and SD thereof. Tentative identifications by one peptide only (see text) are represented as grey lines.

N°	Name	Mr obs.	pI obs.	species	access number	Mr calc.	pI calc.	score	seq.	cov.	rel. Ab.	SD
**Metabolism; Carbohydrate metabolism; Amino sugar and nucleotide sugar metabolism**											
79	fumarylacetoacetate hydrolase, similar	46744	5.76	*Trichoplax adhaerens*	gi|195998011	46138	5.89	85	2	5	0.86	0.08
125	GDP-L-fucose synthetase	32148	8.21	*Crassostrea gigas*	gi|405958300	35147	6.41	76	3	8	1.77	0.21
172	glucosamine phosphate isomerase	24602	7.51	*Idiogaryops pumilis*	gi|262304349	19748	5.39	66	2	13	1.91	0.18
103	UDP-glucose 4-epimerase	38721	6.31	*Crassostrea gigas*	gi|405968861	37674	6.72	113	4	14	0.92	0.13
34	UDP-N-acetylglucosamine pyrophosphorylase, provisional	60863	5.97	*Capitella teleta*	gi|443696999	57560	6.14	99	2	5	0.47	0.05
42	UDP-N-acetylglucosamine pyrophosphorylase, provisional	57755	6.18	*Capitella teleta*	gi|443696999	57560	6.14	98	2	5	0.67	0.12
**Metabolism; Carbohydrate metabolism; Glycolysis / Gluconeogenesis**												
74	enolase	49971	5.84	*Tomocerus sp. jcrjws1*	gi|8101744	41585	5.37	190	4	16	4.02	0.29
93	fructose-bisphosphate aldolase	41755	5.69	*Crassostrea gigas*	gi|405964948	43741	5.88	131	2	8	0.87	0.06
104	fructose-bisphosphate aldolase	37405	6.34	*Mytilus edulis*	gi|46909221	21776	5.86	186	4	23	0.97	0.07
122	glyceraldehyde-3-phosphate dehydrogenase	35098	8.37	*Crassostrea gigas*	gi|405957058	36402	6.95	195	4	9	4.78	0.37
156	glyceraldehyde-3-phosphate dehydrogenase A (EC 1.2.1.12)	27219	6.91	*Urticina eques*	gi|124264159	32082	6.51	70	2	8	3.96	0.18
37	NADPH-dependent aldehyde reductase, putative	60059	4.94	*Mytilus galloprovincialis*	FL493052	29121	5.54	135	3	16	2.71	0.19
27	phosphoenolpyruvate carboxykinase	67990	7.12	*Loa loa*	gi|312080904	72497	6.52	76	4	4	1.03	0.14
28	phosphoenolpyruvate carboxykinase	67990	7.22	*Loa loa*	gi|312080904	72497	6.52	76	4	4	1.21	0.14
101	phosphoglycerate kinase	43890	6.85	*Caenorhabditis brenneri*	gi|341896690	44295	6.28	291	6	18	2.30	0.31
84	phosphoglycerate kinase	43890	7.41	*Crassostrea gigas*	gi|405963233	44217	7.59	85	4	13	1.18	0.11
163	phosphoglycerate mutase 1	25755	4.55	*Pelodictyon phaeoclathratiforme*	Q3VP85_9CHLB	28466	5.20	72	2	8	2.90	0.38
159	triosephosphate isomerase	25844	6.75	*Mytilus edulis*	gi|46909461	16417	4.93	233	5	33	1.88	0.14
157	triosephosphate isomerase, partial	25666	6.04	*Mytilus edulis*	gi|46909461	16417	4.93	330	6	31	1.74	0.07
**Metabolism; Carbohydrate metabolism; Citrate cycle (TCA cycle)**												
10	aconitase 2, mitochondrial isoform 2, similar	83797	6.8	*Strongylocentrotus purpuratus*	gi|115735566	65256	4.96	105	3	6	0.70	0.07
11	aconitase 2, mitochondrial isoform 2, similar	83797	6.97	*Strongylocentrotus purpuratus*	gi|115936456	84808	5.49	219	4	7	0.64	0.09
82	citrate synthase, mitochondrial, predicted	45275	6.49	*Strongylocentrotus purpuratus*	gi|390339579	51662	6.09	101	3	7	1.67	0.17
54	dihydrolipoamide dehydrogenase	52991	6.55	*Trichoplax adhaerens*	gi|196005079	48079	6.74	105	2	5	1.35	0.14
98	isocitrate dehydrogenase	42584	6.36	*Crassostrea gigas*	gi|48476117	51365	8.52	245	5	14	1.17	0.11
99	isocitrate dehydrogenase	42584	6.47	*Crassostrea gigas*	gi|48476117	51365	8.52	216	7	17	0.52	0.04
100	isocitrate dehydrogenase	42584	6.60	*Crassostrea gigas*	gi|48476117	51365	8.52	445	9	20	2.94	0.21
161	isocitrate dehydrogenase	24686	6.88	*Mytilus trossulus*	gi|385268539	50918	6.77	63	2	5	1.70	0.21
171	isocitrate dehydrogenase	25226	6.88	*Mytilus trossulus*	gi|385268539	50918	6.77	63	2	5	2.21	0.18
136	malate dehydrogenase, cytosolic	30138	6.33	*Mytilus galloprovincialis*	gi|73656337	36628	6.02	222	6	24	2.37	0.23
119	malate dehydrogenase, mitochondrial	34527	6.48	*Candida albicans*	gi|68466091	34821	5.73	68	3	9	1.95	0.08
121	malate dehydrogenase, mitochondrial	34527	6.80	*Crassostrea gigas*	gi|405963427	30046	8.20	64	2	7	0.62	0.06
118	malate deshydrogenase, cytosolic	35390	6.07	*Mytilus galloprovincialis*	gi|73656337	36628	6.02	869	16	49	2.44	0.18
113	pyruvate dehydrogenase E1 component subunit beta, mitochondrial	32652	5.39	*Ascaris suum*	gi|129066	39681	5.84	108	3	8	1.62	0.16
**Metabolism; Carbohydrate metabolism; Pentose phosphate pathway **												
36	transketolase	62469	6.90	*Strongylocentrotus purpuratus*	gi|336455050	67029	5.96	118	3	5	0.61	0.08
**Metabolism; Energy metabolism; Transferring phosphorus-containing groups**												
107	arginine kinase	36605	7.29	*Macrobiotus occidentalis*	gi|308199061	40207	6.91	89	2	8	2.44	0.17
108	arginine kinase	36605	7.68	*Conus novaehollandiae*	gi|301341836	39664	6.34	123	2	3	3.95	0.43
**Metabolism; Energy metabolism; Oxidative phosphorylation**												
64	ATP synthase alpha subunit mitochondrial	51140	6.84	*Crassostrea gigas*	gi|405974703	60000	8.48	505	11	18	1.56	0.12
65	ATP synthase alpha subunit mitochondrial	50843	6.92	*Litopenaeus vannamei*	gi|288816877	59416	8.97	284	8	12	2.50	0.35
66	ATP synthase alpha subunit mitochondrial	50549	7.11	*Pinctada fucata*	gi|116008297	59814	8.92	764	14	23	3.29	0.31
87	ATP synthase beta subunit	46535	4.90	*Mytilus edulis*	gi|46909261	46288	4.97	885	16	53	1.94	0.14
152	ETF beta-like	27504	6.02	*Nasonia vitripennis*	gi|156543370	27498	7.66	236	6	19	1.11	0.07
67	NADH dehydrogenase (ubiquinone) flavoprotein 1, mitochondrial	48850	8.37	*Crassostrea gigas*	gi|405967555	51955	8.39	171	5	11	1.01	0.17
164	NADH dehydrogenase [ubiquinone] iron-sulfur protein 8, mitochondrial-like	24770	4.83	*Metaseiulus occidentalis*	gi|391342248	24721	5.42	62	2	10	0.86	0.05
14	NADH dehydrogenase subunit, hypothetical protein DAPPUDRAFT_192333	75534	5.38	*Daphnia pulex*	gi|321476647	80103	6.00	162	3	4	0.55	0.05
15	NADH-ubiquinone oxidoreductase 75 kDa subunit, mitochondrial	75534	5.33	*Crassostrea gigas*	gi|405977043	81477	5.84	180	5	6	0.28	0.03
188	nucleoside diphosphate kinase	17020	7.99	*Ostrea edulis*	gi|388571212	18860	6.82	65	2	12	4.98	0.89
22	succinate dehydrogenase (ubiquinone) flavoprotein subunit	67990	5.59	*Clonorchis sinensis*	gi|358254399	72276	7.09	185	3	5	0.97	0.10
83	succinate-semialdehyde dehydrogenase, mitochondrial	45998	6.92	*Mytilus californianus*	GE753097	29091	8.82	84	2	8	1.41	0.48
167	ubiquinol-cytochrome c reductase, Rieske iron-sulfur polypeptide 1	24770	5.37	*Mytilus galloprovincialis*	FL489022	22838	9.08	283	5	30	1.53	0.15
149	voltage-dependent anion selective channel protein 2, probable	28679	8.24	*Mytilus californianus*	GE752193	23286	5.38	164	3	17	6.18	0.61
**Metabolism; Lipid metabolism**												
154	enoyl-CoA hydratase, mitochondrial-like	27314	6.57	*Amphimedon queenslandica*	gi|340375594	31912	5.82	74	2	10	1.27	0.07
155	enoyl-CoA hydratase, mitochondrial-like	27314	6.68	*Amphimedon queenslandica*	gi|340375594	31912	5.82	100	2	10	1.54	0.08
165	fatty acid-binding protein, provisional	24021	5.06	*Mytilus galloprovincialis*	FL498602	21271	8.51	171	4	33	3.69	0.25
111	inorganic pyrophosphatase-like	33434	5.17	*Mytilus californianus*	ES407080	41244	8.71	152	3	7	1.63	0.11
97	long-chain specific acyl-CoA dehydrogenase, mitochondrial precursor	40566	6.17	*Homo sapiens*	gi|4501857	48024	7.68	90	2	6	0.70	0.08
**Metabolism; Amino acid metabolism**												
3	glycine dehydrogenase	100445	6.12	*Mytilus galloprovincialis*	FL490887	29626	8.23	84	2	10	0.47	0.05
30	delta-1-pyrroline-5-carboxylate dehydrogenase, mitochondrial	63776	5.27	*Crassostrea gigas*	gi|405978465	64148	8.35	74	2	3	0.54	0.03
57	amine oxidase, predicted	54915	7.44	*Nematostella vectensis*	gi|156382450	58581	6.54	54	2	4	0.76	0.09
60	procollagen-proline dioxygenase beta subunit	55950	4.62	*Mytilus galloprovincialis*	gi|390979785	55402	4.53	449	13	25	7.01	0.42
95	glutamine synthetase	42166	5.98	*Tegillarca granosa*	gi|306489668	41952	5.63	203	4	12	1.43	0.08
106	cystathionine gamma-lyase	39809	6.80	*Capitella teleta*	gi|443685366	43775	6.14	78	2	4	1.23	0.08
123	3-hydroxyanthranilate 3,4-dioxygenase	32399	6.70	*Suberites domuncula*	gi|18076468	32433	5.57	70	2	5	0.77	0.07
**Metabolism; Metabolism of other amino-acids**												
135	S-formylglutathione hydrolase	29472	6.25	*Acromyrmex echinatior*	gi|332027837	18955	6.58	138	2	9	1.03	0.08
**Metabolism; Glycan biosynthesis and metabolism**												
112	short chain collagen C4, putative	32148	5.20	*Mytilus galloprovincialis*	EH 663252	32880	8.72	373	7	34	1.15	0.12
**Metabolism; Metabolism of cofactors and vitamins; Ubiquinone and other terpenoid-quinone biosynthesis**												
47	ubiquinone biosynthesis monooxygenase COQ6	58506	6.53	*Harpegnathos saltator*	gi|307192550	52851	8.79	66	2	2	0.68	0.07
193	ubiquinone biosynthesis monooxygenase COQ6	15255	7.27	*Harpegnathos saltator*	gi|307192550	52851	8.79	59	2	2	4.38	1.35
**Genetic Information Processing; Transcription**												
114	transcriptional activator protein pur-alpha	33170	5.45	*Crassostrea gigas*	gi|405974727	27930	6.78	265	6	23	1.13	0.06
115	pur-alpha, putative	33170	5.55	*Ixodes scapularis*	gi|242046488	26667	9.41	111	2	11	1.56	0.11
**Genetic Information Processing; Translation**												
86	40S ribosomal prot SA (p 40) (34/67 kDa laminin receptor)	43011	4.69	*Pinctada fucata*	gi|229891605	33727	5.24	185	4	12	1.73	0.13
183	eIF5A like	17469	5.35	*Mytilus galloprovincialis*	AJ516752	19880	5.23	226	5	38	6.48	0.79
51	elongation factor 1 alpha	56303	5.51	*Mytilus edulis*	gi|299474235	50827	9.12	174	5	14	0.56	0.07
71	elongation factor 1 alpha 1	49971	5.53	*Saccoglossus kowalevskii*	gi|296317283	50711	9.34	134	3	7	1.75	0.24
160	Hadh2-prov protein isoform 1, similar	26477	6.91	*Strongylocentrotus purpuratus*	gi|72006882	27479	6.32	87	2	10	4.13	0.15
25	phenylalanyl-tRNA synthetase beta chain, probable	67990	5.92	*Mytilus galloprovincialis*	FL494288	25820	5.63	138	4	17	0.84	0.07
26	phenylalanyl-tRNA synthetase beta chain, probable	67990	6.15	*Mytilus galloprovincialis*	FL494288	25820	5.63	99	2	9	0.94	0.12
46	PRP19/PSO4 pre-mRNA processing factor 19 homolog, predicted	57755	6.43	*Saccoglossus kowalevskii*	gi|291228334	56436	6.60	78	2	6	0.49	0.04
199	ribosomal protein rps12	13157	5.94	*Lineus viridis*	gi|166952363	13852	8.13	119	5	29	3.39	0.60
185	ribosomal protein rps13	16142	5.11	*Arenicola marina*	gi|158187708	17169	10.59	74	2	17	1.74	0.16
198	ribosomal protein S12	13258	5.62	*Periplaneta americana*	gi|21217441	15585	5.95	106	3	15	4.24	0.37
137	ribosomal protein S2	30138	6.48	*Chlamys farreri*	gi|22203717	27078	10.49	147	5	26	0.65	0.07
**Genetic Information Processing; Folding, sorting and degradation; Folding and sorting**												
13	78kDa glucose regulated protein	75534	4.87	*Crassostrea gigas*	gi|46359618	73088	5.02	567	11	16	3.93	0.36
68	calreticulin, predicted	50750	4.76	*Mytilus galloprovincialis*	FL593839	27230	5.24	564	12	44	8.28	0.36
85	calumenin precursor, putative	44572	4.76	*Pediculus humanus corporis*	gi|242005220	37885	4.61	65	2	3	3.60	0.20
38	chaperonin	56660	5.18	*Paracentrotus lividus*	gi|5912574	62195	5.12	203	4	11	3.67	0.47
146	endoplasmic reticulum protein ERp29	28479	5.85	*Crassostrea gigas*	gi|405975720	28444	5.19	141	3	8	1.09	0.13
4	glucose-regulated protein 94	95115	4.88	*Crassostrea gigas*	gi|148717303	91795	4.83	384	8	10	1.60	0.18
186	glucose-regulated protein 94 (fragment)	16505	5.19	*Crassostrea gigas*	gi|148717303	91795	4.83	101	2	3	1.25	0.08
20	heat shock cognate 71	68990	5.25	*Mytilus galloprovincialis*	gi|76780612	71508	5.29	1515	28	46	4.93	0.23
39	heat shock protein 60	60059	5.23	*Biomphalaria glabrata*	gi|218683627	31076	5.41	400	8	12	1.02	0.17
23	heat shock protein 70	67013	5.64	*Mytilus galloprovincialis*	gi|62989584	68848	5.35	90	3	6	0.75	0.09
24	heat shock protein 70	67013	5.71	*Mytilus galloprovincialis*	gi|62989584	69848	5.35	238	5	8	0.82	0.12
12	heat shock protein 90	81772	5.54	*Mytilus galloprovincialis*	gi|205362524	83358	4.85	179	4	7	0.45	0.07
75	NFX1-type containing zinc finge, similar	51140	5.99	*Hydra magnipapillata*	gi|221116469	395486	8.08	59	3	0	3.20	1.15
143	prohibitin	27504	5.38	*Trichinella spiralis*	gi|339249751	60213	6.90	129	4	6	2.40	0.17
70	protein disulfide-isomerase, like	50843	5.42	*Mytilus californianus*	GE750884	30856	5.07	198	4	19	1.54	0.09
55	protein disulfide-isomerase, predicted	52991	6.64	*Trichoplax adhaerens*	gi|196002337	52300	8.18	76	2	5	1.14	0.08
166	putative small 22kd heat shock protein	24770	5.35	*Mytilus californianus*	ES737901	25707	5.94	80	2	11	1.39	0.11
168	small 22kd heat shock protein, putative	24518	5.49	*Mytilus californianus*	ES737901	25707	5.94	80	2	11	1.98	0.13
131	small heat shock protein 24.1	29692	5.4	*Mytilus galloprovincialis*	gi|347545633	28691	5.61	163	3	12	1.42	0.12
132	small heat shock protein 24.1	29582	5.54	*Mytilus galloprovincialis*	gi|347545633	28691	5.61	200	4	15	2.34	0.27
133	small heat shock protein 24.1	29582	5.73	*Mytilus galloprovincialis*	gi|347545633	28691	5.61	534	12	48	2.57	0.20
144	Small heat shock protein 24.1	28479	5.52	*Mytilus galloprovincialis*	gi|347545633	28691	5.61	75	2	9	3.71	0.34
145	small heat shock protein 24.1	28881	5.64	*Mytilus galloprovincialis*	gi|347545633	28691	5.61	84	2	8	2.18	0.45
148	small heat shock protein 24.1	28280	6.17	*Mytilus galloprovincialis*	gi|347545633	28691	5.61	96	2	8	0.93	0.07
19	stress-70 protein, mitochondrial, predicted mortaline-like	67499	5.21	*Strongylocentrotus purpuratus*	gi|72014569	76579	5.51	264	6	8	1.06	0.15
56	TCP1 subunit epsilon like, hypothetical protein SINV_10604	54915	7.29	*Solenopsis invicta*	gi|322800807	59845	5.80	172	4	9	0.77	0.08
48	TCP1 subunit zeta	57755	6.77	*Haliotis discus hannai*	gi|379318220	58706	6.53	186	4	12	0.66	0.06
45	TCP1, hypothetical protein	58506	6.37	*Amblyomma maculatum*	gi|346470969	59522	5.96	333	8	16	0.84	0.13
33	TCP1, subunit beta-like	61272	5.69	*Saccoglossus kowalevskii*	gi|291227173	150827	8.07	148	4	3	1.05	0.07
44	TCP1, subunit gamma isoform 1	60059	6.29	*Strongylocentrotus purpuratus*	gi|115711990	60965	7.85	120	4	7	0.58	0.06
43	TCP1, subunit eta-like isoform 1	55950	6.29	*Bombus terrestris*	gi|340715736	60400	6.22	193	3	7	0.56	0.05
40	TCP1, subunit theta	58128	5.59	*Crassostrea gigas*	gi|405961548	83831	5.67	175	4	6	0.70	0.06
180	translationally controlled tumour protein	20172	5.28	*Mytilus californianus*	gi|359359687	19635	4.76	71	2	15	1.43	0.24
187	tubulin-specific chaperone a-like	16150	5.65	*Mytilus californianus*	ES738008	26274	6.17	147	4	19	2.87	0.22
5	valosin-containing protein-like	93484	5.14	*Saccoglossus kowalevskii*	gi|291242207	90395	5.18	296	6	10	0.87	0.10
**Genetic Information Processing; Folding, sorting and degradation; Proteasome**												
76	26S protease regulatory subunit 6a RPT5	48040	5.09	*Crassostrea gigas*	gi|405957859	48206	5.08	303	5	14	0.86	0.17
77	26S protease regulatory subunit 6a RPT5	47776	5.18	*Aedes aegypti*	gi|157129681	47953	5.20	269	6	17	1.71	0.17
96	26S proteasome regulatory complex ATPase RPT4	42584	6.12	*Daphnia pulex*	gi|321461635	44199	6.10	291	6	22	1.04	0.09
69	26S proteasome regulatory subunit T3	49405	5.21	*Schistosoma japonicum*	gi|226471414	46930	5.29	563	13	32	0.85	0.10
17	E3 ubiquitin-protein ligase TRIM33	75534	6.05	*Mytilus galloprovincialis*	AJ625521	20697	7.07	174	4	24	0.26	0.04
120	proteasome 26S subunit, non-ATPase 14-like, predicted	33836	6.69	*Saccoglossus kowalevskii*	gi|291239801	34852	6.07	89	3	9	0.82	0.09
142	proteasome alpha 5 subunit-like	27889	4.88	*Saccoglossus kowalevskii*	gi|291243435	26525	4.74	268	4	22	1.24	0.07
169	proteasome alpha type 2	24435	5.79	*Haliotis discus discus*	gi|126697376	26249	5.73	173	3	18	1.35	0.10
177	proteasome beta type-6 subunit	22320	6.33	*Mytilus californianus*	ES387982	30469	7.13	421	9	43	1.15	0.12
147	proteasome subunit alpha type-4	28280	6.04	*Crassostrea gigas*	gi|405964515	21464	5.69	70	2	13	1.26	0.17
153	proteasome subunit alpha type-6	27600	6.12	*Crassostrea gigas*	gi|405975869	25429	7.57	182	4	18	1.13	0.10
176	ubiquination linked effector, hypothetical protein CRE_31518	22248	6.26	*Caenorhabditis remanei*	gi|308460407	37338	8.82	57	*1*	2	0.97	0.08
**Genetic Information Processing; Replication and repair**												
197	histone H2B	13942	5.23	*Mytilus edulis*	gi|23304756	13781	10.69	91	2	19	2.35	0.22
202	histone H4	12350	5.76	*Diprion pini*	gi|1883030	11141	11.51	79	3	32	1.17	0.08
41	meiosis-specific nuclear structural protein 1-like	57021	5.91	*Saccoglossus kowalevskii*	gi|291241736	61112	5.52	107	3	3	0.76	0.09
**Environmental Information Processing; Signal transduction**												
128	14-3-3 epsilon protein	29364	4.71	*Bombyx mori*	gi|148298752	29767	4.66	267	7	24	1.55	0.14
130	14-3-3 epsilon protein	29364	4.95	*Lepeophtheirus salmonis*	gi|155966250	28466	4.67	102	3	8	3.44	0.27
129	14-3-3 epsilon protein	28980	4.69	*Bombyx mori*	gi|148298752	29767	4.66	214	7	24	2.07	0.28
174	calcyphosin-like protein	24505	5.61	*Mytilus galloprovincialis*	FL489968	22644	7.00	141	2	10	1.64	0.28
18	EF-hand domain-containing protein 1	72142	6.66	*Crassostrea gigas*	gi|405964721	74735	6.23	126	4	5	1.10	0.08
**Environmental Information Processing; Signaling molecules and interaction**												
182	cyclophilin-type peptidyl-prolyl cis-trans isomerase-15	21545	5.95	*Mytilus galloprovincialis*	FL494508	21737	5.77	305	6	28	1.22	0.26
32	dedicator of cytokinesis protein 8, partial, predicted	61686	5.57	*Amphimedon queenslandica*	gi|340379755	236465	6.29	53	2	2	0.68	0.06
116	G protein subunit beta-1	34110	5.65	*Loligo forbesii*	gi|121014	37983	5.76	403	9	34	1.12	0.08
162	GTP-binding nuclear protein Ran, provisional	25139	7.99	*Crassostrea gigas*	gi|405971745	24274	6.96	72	2	10	3.01	0.32
195	peptidyl prolyl cis-trans isomerase A (II)	14887	8.55	*Conus novaehollandiae*	gi|289064183	17759	7.68	178	4	20	6.17	0.75
138	receptor of Activated Kinase C 1	30251	6.96	*Mya arenaria*	gi|115501910	35534	6.74	233	7	30	0.88	0.06
139	receptor of Activated Kinase C 1	30251	7.29	*Mya arenaria*	gi|115501910	35534	6.74	662	15	58	2.24	0.10
140	receptor of Activated Kinase C 1	30251	7.41	*Mya arenaria*	gi|115501910	35534	6.74	510	12	41	1.03	0.07
80	RIB43A-like with coiled-coils protein 2	46744	6.04	*Crassostrea gigas*	gi|405963849	45583	6.09	124	4	5	1.64	0.34
31	serine/threonine-protein kinase pelle-like	63776	5.44	*Bombus impatiens*	gi|350396247	58945	8.87	54	*1*	1	1.01	0.10
102	SET protein	37354	4.49	*Crassostrea gigas*	gi|405963180	28144	4.34	197	4	12	2.76	0.25
124	sirtuin-5	32399	7.05	*Aplysia californica*	gi|325197143	39468	9.03	117	2	6	0.82	0.07
**Cellular Processes; Transport and catabolism**												
181	C1q domain containing protein MgC1q64, putative	20473	5.85	*Mytilus galloprovincialis*	gi|325504427	24551	8.32	65	2	20	0.85	0.09
59	catalase	53914	7.99	*Mytilus californianus*	gi|46909299	30345	6.01	235	7	34	1.31	0.28
141	cathepsin L-like, predicted	27219	4.55	*Strongylocentrotus purpuratus*	gi|115715524	37335	5.14	64	2	3	2.23	0.23
16	dipeptidyl peptidase family member 6	72142	5.68	*Crassostrea gigas*	gi|405969597	74497	5.66	60	*1*	1	0.46	0.03
134	dyp-type peroxidase like	30364	6.00	*Trichoplax adhaerens*	gi|195996389	33144	6.21	59	2	6	1.03	0.11
173	glutathione S-transferase sigma 3	22964	5.56	*Mytilus galloprovincialis*	gi|402227995	22940	5.44	121	3	18	0.65	0.06
158	glutathione S-transferase, Class Beta	25489	6.57	*Mytilus californianus*	ES392983	38159	5.76	74	2	8	1.96	0.15
110	heavy metal-binding protein HIP	34810	4.92	*Mytilus edulis*	gi|46395578	24388	5.09	165	6	45	2.43	0.38
105	kin 17-mid super family, hypothetical protein AND_04962	38721	6.53	*Anopheles darlingi*	gi|312382372	48048	9.44	55	2	5	0.57	0.04
58	leucine aminopeptidase, predictive	54915	8.03	*Mytilus californianus*	ES400183	36649	7.01	332	8	40	2.32	0.25
6	major vault protein	91110	5.48	*Mytilus edulis*	gi|5714749	31855	5.45	343	9	46	0.50	0.07
8	major vault protein	91892	5.55	*Mytilus edulis*	gi|5714749	31855	5.45	718	16	56	1.27	0.12
7	major vault protein	91892	5.53	*Crassostrea gigas*	gi|405974681	96651	5.58	73	2	2	0.79	0.08
9	major vault protein	90338	5.61	*Mytilus edulis*	gi|5714749	31855	5.45	276	8	35	1.07	0.10
170	peroxiredoxin	24186	6.7	*Pinctada fucata*	gi|306451460	22530	7.63	99	2	9	1.81	0.23
184	peroxiredoxin V	17924	6.38	*Chlamys farreri*	gi|149688674	20431	8.20	69	*1*	5	4.55	0.24
81	Rab GDP dissociation inhibitor alpha	45998	6.21	*Schistosoma japonicum*	gi|226484726	50623	6.41	60	2	4	1.32	0.51
190	superoxide dismutase	14887	5.77	*Mytilus chilensis*	gi|332356353	15925	5.84	173	4	30	2.23	0.18
191	superoxide dismutase (Cu/Zn-SOD)	14673	6.11	*Mytilus edulis*	gi|34481600	16046	5.84	289	4	31	3.40	0.22
175	superoxide dismutase, mitochondrial (Mn-SOD)	22327	6.00	*Mytilus galloprovincialis*	gi|402122769	25412	6.44	124	2	9	1.67	0.15
203	thioredoxin 1	12520	4.69	*Mytilus galloprovincialis*	gi|391358072	11667	4.47	244	4	33	7.72	0.72
178	thioredoxin peroxidase	23774	6.70	*Cristaria plicata*	gi|306451460	22143	5.95	75	2	10	1.63	0.16
21	V-type proton ATPase catalytic subunit A	72142	5.45	*Crassostrea gigas*	gi|405950221	71148	5.21	314	7	11	0.69	0.06
**Cellular Processes; Cell motility; Cytoskeleton proteins**												
92	actin	43011	5.60	*Mytilus sp.*	gi|120564812	35392	5.26	93	3	14	0.90	0.10
189	actin	15106	5.15	*Schistosoma japonicum*	gi|257215973	10215	5.40	273	6	55	3.99	0.45
196	actin	14330	5.05	*Hydroides elegans*	gi|73532714	41520	5.39	357	9	19	2.90	0.28
88	actin 2 = cytoplasmic actin = beta actin	42200	5.22	*Crassostrea gigas*	gi|18565104	42002	5.30	669	15	47	5.83	0.44
89	actin 2 = cytoplasmic actin = beta actin	42200	5.31	*Aedes aegypti*	gi|67782283	42194	5.30	648	14	49	15.31	0.97
91	actin 2 = cytoplasmic actin = beta actin	42200	5.4	*Mytilus sp.*	gi|120564812	35392	5.26	454	11	51	1.86	0.13
151	actin 2 = cytoplasmic actin = beta actin	26568	5.49	*Crassostrea gigas*	gi|18565104	42002	5.30	444	11	32	3.25	0.23
94	actin 5	40566	5.73	*Aedes aegypti*	gi|67782283	42194	5.3	404	10	35	1.06	0.10
150	actin-87E isoform 1, similar	26845	5.28	*Tribolium castaneum*	gi|91078486	42158	5.29	419	10	36	5.20	0.59
2	catchin protein	113783	5.32	*Mytilus galloprovincialis*	gi|6682323	112777	5.22	701	16	21	0.40	0.02
179	centrin-3	19785	4.66	*Crassostrea gigas*	gi|405964350	20761	4.58	139	4	22	2.93	0.21
192	destrin, partial	15330	6.38	*Macaca mulatta*	gi|73696362	12274	8.64	64	2	7	7.79	0.54
52	fascin	53914	6.01	*Crassostrea gigas*	gi|405961655	56081	6.21	99	3	5	1.29	0.08
53	fascin-like domain protein	53914	6.15	*Tetraodon nigroviridis*	gi|47209051	106026	8.68	85	2	2	0.74	0.08
78	gelsolin	46245	5.61	*Suberites domuncula*	gi|27528508	42414	5.23	115	2	7	2.17	0.09
194	hypothetical protein KGM_09271 with pleckstrin homology-like domain	14603	7.58	*Danaus plexippus*	gi|357623784	110881	9.64	72	2	2	4.85	0.72
29	Na(+)/H(+) exchange regulatory cofactor NHE-RF1	63335	4.83	*Mytilus galloprovincialis*	FL501152	22127	4.93	347	6	42	1.39	0.37
62	non-neuronal cytoplasmic intermediate filament protein	56303	5.27	*Mytilus californianus*	GE750313	31541	7.63	410	9	29	2.03	0.27
200	profilin like	12468	6.68	*Mytilus galloprovincialis*	FL496207	20580	8.33	243	6	37	5.42	0.37
117	radial spoke head protein 9, like	32909	5.82	*Crassostrea gigas*	gi|405959092	31220	5.20	118	3	8	1.21	0.22
1	spectrin alpha chain	105775	4.83	*Crassostrea gigas*	gi|405973516	287684	4.88	143	5	2	0.44	0.04
90	tektin 1	45755	5.36	*Crassostrea gigas*	gi|405975636	48654	6.12	55	2	3	1.86	0.12
72	tektin-2	49971	5.58	*Crassostrea gigas*	gi|405950079	48059	5.71	172	6	18	2.60	0.14
73	tektin-4	48307	5.64	*Crassostrea gigas*	gi|405967050	52952	5.53	172	7	12	2.72	0.18
109	tropomyosin	35098	4.65	*Mytilus galloprovincialis*	gi|6647862	32807	4.62	559	12	36	5.25	0.23
126	tropomyosin	30478	4.69	*Mytilus edulis*	gi|6647862	32836	4.64	312	6	12	1.81	0.13
127	tropomyosin	30593	4.77	*Mytilus galloprovincialis*	gi|6647862	32807	4.62	190	4	8	2.17	0.08
49	tubulin alpha-1 chain	54915	5.09	*Schistosoma mansoni*	gi|256087763	50660	4.97	780	18	47	11.21	0.75
61	tubulin beta chain	51744	4.93	*Crassostrea gigas*	gi|56603670	50371	4.79	705	15	37	31.68	1.30
63	tubulin, beta 2C-like, predicted	56303	5.40	*Saccoglossus kowalevskii*	gi|291243365	50516	4.74	266	6	16	3.06	0.32
**Unknown function**												
35	CCDC 151 like, coiled-coil domain containing 151	62469	6.68	*Crassostrea gigas*	gi|405957528	63895	6.65	68	2	2	1.21	0.12
50	selenium-binding protein 1, partial	57755	5.48	*Crassostrea gigas*	gi|405971621	54060	6.11	56	2	2	0.67	0.08
201	hypothetical protein AND_08398	12519	7.95	*Anopheles darlingi*	gi|312379666	38819	8.84	53	2	0	4.58	0.55

**Table 2:** Classification of the 203 proteoforms listed in Table 1 according to their coefficient of quartile variation (cqv in %) indicating the spread in relative protein abundance obtained over five runs of 12 gels each (*n* = 60). See text for further explication.

N°	Proteoforme	cqv		
99	isocitrate dehydrogenase	5.9	<10%	
165	fatty acid-binding protein, provisional	6.7		
72	tektin-2	6.8		
192	destrin, partial	7.4		
78	gelsolin	7.7		
73	tektin-4	7.8		
157	triosephosphate isomerase, partial	8.0		
139	receptor of Activated Kinase C 1	8.2		
160	Hadh2-prov protein isoform 1, similar	8.3		
23	heat shock protein 70	8.4		
60	procollagen-proline dioxygenase beta subunit	8.4		
61	tubulin beta chain	8.9		
109	tropomyosin	9.0		
175	superoxide dismutase, mitochondrial (Mn-SOD)	9.1		
68	calreticulin, predicted	9.1		
119	malate dehydrogenase, mitochondrial	9.2		
74	enolase	9.5		
127	tropomyosin	9.5		
156	glyceraldehyde-3-phosphate dehydrogenase A (EC 1.2.1.12)	9.6		
104	fructose-bisphosphate aldolase	9.7		
144	Small heat shock protein 24.1	9.9		
150	actin-87E isoform 1, similar	10.2	<15%	
105	kin 17-mid super family, hypothetical protein AND_04962	10.2		
115	pur-alpha, putative	10.3		
93	fructose-bisphosphate aldolase	10.5		
155	enoyl-CoA hydratase, mitochondrial-like	10.6		
86	40S ribosomal prot SA (p 40) (34/67 kDa laminin receptor)	10.7		
143	prohibitin	10.9		
142	proteasome alpha 5 subunit-like	11.0		
16	dipeptidyl peptidase family member 6	11.0		
88	actin 2 = cytoplasmic actin= beta actin	11.0		
95	glutamine synthetase	11.0		
106	cystathionine gamma-lyase	11.0		
118	malate deshydrogenase, cytosolic	11.0		
138	receptor of Activated Kinase C 1	11.0		
122	glyceraldehyde-3-phosphate dehydrogenase	11.1		
20	heat shock cognate 71	11.1		
136	malate dehydrogenase, cytosolic	11.5		
89	actin 2 = cytoplasmic actin = beta actin	11.6		
202	histone H4	11.8		
66	ATP synthase alpha subunit mitochondrial	11.9		
168	small 22kd heat shock protein, putative	12.0		
30	delta-1-pyrroline-5-carboxylate dehydrogenase, mitochondrial	12.0		
96	26S proteasome regulatory complex ATPase RPT4	12.0		
185	ribosomal protein rps13	12.2		
70	protein disulfide-isomerase, like	12.2		
82	citrate synthase, mitochondrial, predicted	12.4		
52	fascin	12.4		
35	CCDC 151 like, coiled-coil domain containing 151	12.4		
200	profilin like	12.5		
152	ETF beta-like	12.5		
184	peroxiredoxin V	12.6		
135	S-formylglutathione hydrolase	12.7		
116	G protein subunit beta-1	12.8		
179	centrin-3	12.8		
55	protein disulfide-isomerase, predicted	12.9		
108	arginine kinase	13.0		
128	14-3-3 epsilon protein	13.0		
154	enoyl-CoA hydratase, mitochondrial-like	13.1		
9	major vault protein	13.3		
18	EF-hand domain-containing protein 1	13.3		
113	pyruvate dehydrogenase E1 component subunit beta, mitochondrial	13.3		
203	thioredoxin 1	13.3		
186	glucose-regulated protein 94 (fragment)	13.4		
141	cathepsin L-like, predicted	13.4		
33	TCP1, subunit beta-like	13.4		
85	calumenin precursor, putative	13.4		
111	inorganic pyrophosphatase-like	13.6		
147	proteasome subunit alpha type-4	13.6		
114	transcriptional activator protein pur-alpha	13.6		
53	fascin-like domain protein	13.7		
191	superoxide dismutase (Cu/Zn-SOD)	13.7		
32	dedicator of cytokinesis protein 8, partial, predicted	14.0		
71	elongation factor 1 alpha 1	14.0		
129	14-3-3 epsilon ptotein	14.2		
183	eIF5A like	14.2		
40	TCP1, subunit theta	14.3		
13	78kDa glucose regulated protein	14.4		
126	tropomyosin	14.4		
84	phosphoglycerate kinase	14.7		
90	tektin 1	15.0	<20%	
43	TCP1, subunit eta-like isoform 1	15.1		
130	14-3-3 epsilon protein	15.1		
91	actin 2 = cytoplasmic actin = beta actin	15.2		
25	phenylalanyl-tRNA synthetase beta chain, probable	15.2		
190	superoxide dismutase	15.3		
37	NADPH-dependent aldehyde reductase, putative	15.4		
2	catchin protein	15.4		
159	triosephosphate isomerase	15.5		
197	histone H2B	15.7		
76	26S protease regulatory subunit 6a RPT5	15.9		
194	hypothetical protein KGM_09271 with pleckstrin homology-like domain	15.9		
164	NADH dehydrogenase [ubiquinone] iron-sulfur protein 8, mitochondrial-like	15.9		
158	glutathione S-transferase, Class Beta	16.0		
67	NADH dehydrogenase (ubiquinone) flavoprotein 1, mitochondrial	16.0		
140	receptor of Activated Kinase C 1	16.0		
62	non-neuronal cytoplasmic intermediate filament protein	16.1		
188	nucleoside diphosphate kinase	16.1		
87	ATP synthase beta subunit	16.2		
151	actin 2 = cytoplasmic actin = beta actin	16.5		
132	small heat shock protein 24.1	16.6		
107	arginine kinase	16.9		
94	actin 5	16.9		
8	major vault protein	16.9		
195	peptidyl prolyl cis-trans isomerase A (II)	16.9		
4	glucose-regulated protein 94	17.0		
46	PRP19/PSO4 pre-mRNA processing factor 19 homolog, predicted	17.0		
10	aconitase 2, mitochondrial isoform 2, similar	17.0		
7	major vault protein	17.1		
121	malate dehydrogenase, mitochondrial	17.2		
173	glutathione S-transferase sigma 3	17.3		
187	tubulin-specific chaperone a-like	17.4		
65	ATP synthase alpha subunit mitochondrial	17.4		
14	NADH dehydrogenase subunit, hypothetical protein DAPPUDRAFT_192333	17.5		
79	fumarylacetoacetate hydrolase, similar	17.5		
64	ATP synthase alpha subunit mitochondrial	17.9		
123	3-hydroxyanthranilate 3,4-dioxygenase	17.9		
117	radial spoke head protein 9, like	17.9		
153	proteasome subunit alpha type-6	18.0		
59	catalase	18.1		
80	RIB43A-like with coiled-coils protein 2	18.2		
189	actin	18.2		
44	TCP1, subunit gamma isoform 1	18.4		
100	isocitrate dehydrogenase	18.4		
48	TCP1 subunit zeta	18.4		
171	isocitrate dehydrogenase	18.5		
3	glycine dehydrogenase	18.5		
177	proteasome beta type-6 subunit	18.5		
178	thioredoxin peroxidase	18.5		
45	TCP1, hypothetical protein	18.7		
131	small heat shock protein 24.1	19.2		
69	26S proteasome regulatory subunit T3	19.3		
21	V-type proton ATPase catalytic subunit A	19.3		
133	small heat shock protein 24.1	19.3		
149	voltage-dependent anion selective channel protein 2, probable	19.6		
102	SET protein	19.6		
12	heat shock protein 90	19.6		
41	meiosis-specific nuclear structural protein 1-like	19.7		
148	small heat shock protein 24.1	19.8		
174	calcyphosin-like protein	19.8		
198	ribosomal protein S12	19.9		
176	ubiquination linked effector, hypothetical protein CRE_31518	20.0	<25%	
49	tubulin alpha-1 chain	20.2		
17	E3 ubiquitin-protein ligase TRIM33	20.3		
103	UDP-glucose 4-epimerase	20.3		
77	26S protease regulatory subunit 6a RPT5	20.4		
19	stress-70 protein, mitochondrial, predicted mortaline-like	20.6		
22	succinate dehydrogenase (ubiquinone) flavoprotein subunit	20.6		
172	glucosamine phosphate isomerase	20.6		
27	phosphoenolpyruvate carboxykinase	20.6		
124	sirtuin-5	20.6		
15	NADH-ubiquinone oxidoreductase 75 kDa subunit, mitochondrial	20.8		
169	proteasome alpha type 2	20.8		
199	ribosomal protein rps12	20.9		
134	dyp-type peroxidase like	21.0		
98	isocitrate dehydrogenase	21.0		
47	ubiquinone biosynthesis monooxygenase COQ6	21.0		
29	Na(+)/H(+) exchange regulatory cofactor NHE-RF1	21.1		
170	peroxiredoxin	21.1		
196	actin	21.1		
166	putative small 22kd heat shock protein	21.2		
181	C1q domain containing protein MgC1q64, putative	21.2		
162	GTP-binding nuclear protein Ran, provisional	21.5		
57	amine oxidase, predicted	21.5		
39	heat shock protein 60	21.7		
31	serine/threonine-protein kinase pelle-like	21.8		
56	TCP1 subunit epsilon like, hypothetical protein SINV_10604	22.1		
36	transketolase	22.1		
97	long-chain specific acyl-CoA dehydrogenase, mitochondrial precursor	22.4		
63	tubulin, beta 2C-like, predicted	22.5		
182	cyclophilin-type peptidyl-prolyl cis-trans isomerase-15	22.5		
112	short chain collagen C4, putative	22.6		
1	spectrin alpha chain	22.8		
42	UDP-N-acetylglucosamine pyrophosphorylase, provisional	23.1		
34	UDP-N-acetylglucosamine pyrophosphorylase, provisional	23.2		
146	endoplasmic reticulum protein ERp29	23.4		
193	ubiquinone biosynthesis monooxygenase COQ6	23.5		
28	phosphoenolpyruvate carboxykinase	23.8		
38	chaperonin	24.1		
167	ubiquinol-cytochrome c reductase, Rieske iron-sulfur polypeptide 1	24.1		
58	leucine aminopeptidase, predictive	24.6		
50	selenium-binding protein 1, partial	24.7		
5	valosin-containing protein-like	24.8		
51	elongation factor 1 alpha	25.9	<30%	
137	ribosomal protein S2	25.9		
163	phosphoglycerate mutase 1	25.9		
92	actin	26.4		
26	phenylalanyl-tRNA synthetase beta chain, probable	26.9		
54	dihydrolipoamide dehydrogenase	27.1		
101	phosphoglycerate kinase	27.6		
201	hypothetical protein AND_08398	27.7		
125	GDP-L-fucose synthetase	28.0		
145	small heat shock protein 24.1	31.4	>30%	
24	heat shock protein 70	33.5		
81	Rab GDP dissociation inhibitor alpha	37.1		
11	aconitase 2, mitochondrial isoform 2, similar	37.4		
180	translationally controlled tumour protein	40.8		
83	succinate-semialdehyde dehydrogenase, mitochondrial	43.8		
75	NFX1-type containing zinc finge, similar	47.0		

**Figure 3:**
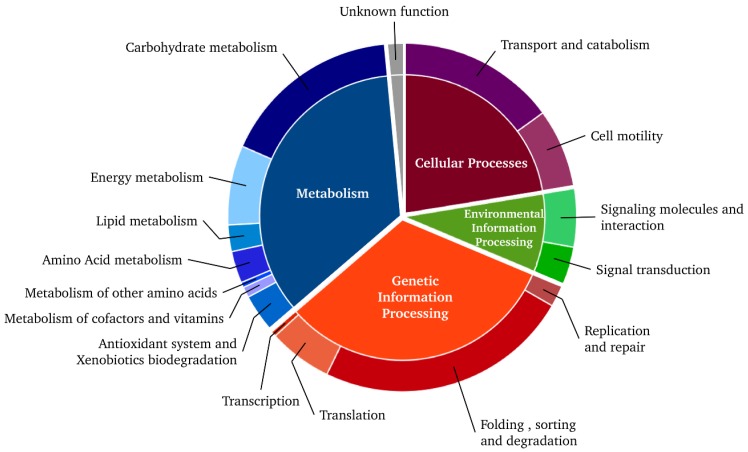
Functional classification of the 203 protein spots identified for *Mytilus edulis* gills according to their metabolic pathways and cellular functions (KEGG). Data derived from Table 1.

In the following sections, proteoforms that have yielded identification were grouped and will be discussed according to their principal cellular functions derived from the KEGG pathway classification as depicted in Figure 3. It becomes obvious, that a great number of the identified proteins either (*i*) belong to the cytoskeleton; or (*ii*) are involved in protein synthesis and degradation; or (*iii*) have key functions in the energetic metabolism and cellular defence. Interestingly, several of these highly expressed proteins exhibit characteristics that reflect specificities of the organization and function of the mussel gill tissue.

### 3.1. Transcriptional and Translational Actors

Classical ribosome-associated proteins are found abundantly in the blue mussel gill proteome (e.g., spot no. 25, 26, 86, 137, 185, 198 and 199). In addition, two ubiquitous and highly conserved translation factors were identified, namely, eukaryotic initiation factor 5A (eIF5A, spot no. 183) and eukaryotic elongation factor 1 alpha 1 (eEF1α1, spot no. 71). Interestingly, the expression of these factors is redox sensitive [66,67]. The factor eEF1α is one of the most abundant cytoplasmic proteins and is responsible for the binding of aminoacyl-tRNA to the ribosome in a GTP-dependent process [68]. It is also involved in the ubiquitination of proteins [69]. Interestingly, we identified the Pur-alpha protein (spot no. 114 and 115), a single-stranded DNA-binding protein implicated in the control of both DNA replication and gene transcription [70]. Pur-alpha may interact with E2F1, a DNA-binding transcription factor, which is known to play a role in ROS accumulation via the NF-kappa-B/MnSOD signal pathway related to oxidative stress [71].

### 3.2. Cytoskeleton

As expected from the structural organization of the mussel gills, characterised by ciliated filaments, actins and tubulins constitute the quantitatively most abundant proteins of this tissue (spot no. 49, 61, 89). Other cytoskeleton components are also well-represented as demonstrated by the identification of intermediate filament proteins (spot no. 2: catchin; spot no. 62: non-neuronal cytoplasmic intermediate filament protein) and a number of actin binding proteins (ABPs) (spot 1: spectrin; spot no. 52: fascin; spot no. 78: gelsolin; spots no. 109, no. 126 and 127: tropomyosin; spot no. 192: destrin; spot no. 200: profiling-like; spot no. 194: pleckstrin-like). Among them, notably non-muscular tropomyosins are involved in a range of cellular functions that control and regulate the cells cytoskeleton. Studies suggest that the binding of tropomyosin isoforms to an actin filament may influence the binding of other ABPs, which together alter the structure and endow specific properties and functions to an actin filament [72]. Among them, profilin (spot no. 200) is an ABP involved in the dynamic turnover and restructuring of the actin cytoskeleton. Gelsolin (spot no. 78) acts also as a key regulator of actin filament assembly and disassembly. Numerous other identified proteins also potentially interact with the actin and tubulin networks. For example, the dedicator of cytokinesis 8 (spot no. 32) is implicated in the regulation of the actin cytoskeleton. Proteins containing long coiled-coil domains like the RIB43A-like with coiled-coils protein 2 (spot no. 80) are involved in tying other proteins to solid-state components of the cell [73].

Several protein identifications point to the particular nature of a ciliated epithelial structure, which is characterized by a specific organisation of actin microfilaments and tubulin microtubules. Tektins (spot no. 72, 73 and no. 90) are cytoskeletal proteins found in cilia and flagella as structural components of outer doublet microtubules. Radial spoke head proteins 9 (spot no. 117) are involved in the movement of cilia and consist of (*i*) a thin stalk, which is attached to a subfiber of the outer doublet microtubule, and (*ii*) a bulbous head, which is attached to the stalk and interacts with the projections from the central pair of microtubules [74]. The Na(+)H(+) exchange regulatory cofactor NHE-RF1 (spot no. 29), also named Ezrin-radixin-moesin (ERM)-binding phosphoprotein 50, helps to link members of the ERM family to the actin cytoskeleton as well as to regulate their surface expression. The ERM proteins are highly concentrated in the apical part of polarized epithelial cells and are thought to be linkers between integral membrane and cytoskeletal proteins [75]. We also identified SET (spot no. 102), a phosphatase inhibitor 2, which is a multifunctional protein that, amongst other functions, regulates the microtubule networks of cilia. For instance, in primary cilia of human renal epithelium cells, endogenous phosphatase inhibitor 2 was found to be highly expressed and involved in the early formation of cilia [76].

### 3.3. Energetic, Carbohydrate and Amino Acid Metabolisms

The relatively high number of mitochondrial proteins related to energetic metabolism is consistent with the supposedly high energetic demand of gill tissue, which is engaged in water movement and transport of food particles. Also, osmoregulatory ion-transport via the gill epithelia is likely to be coupled to oxidative metabolism [77,78]. Furthermore, the function of chaperones and the proteasome (see sections below) depend on ATP-cycling [79,80]. Major carbohydrate metabolic pathways are represented by six enzymes of the tricarboxylic acid cycle (spot no. 11, 54, 82, 100, 113 and 119) and numerous enzymes of the oxidative phosphorylation process (for example, spot no. 66: ATP synthase alpha subunit; spot no. 87: ATP synthase beta subunit; spot no. 14: NADH dehydrogenase subunit). Glycolytic enzymes of the cytosol are also well represented with nine enzymes implied in glycolysis/neoglucogenesis. A prominent example is that of arginine kinase (spot no. 107 and 108), which plays an important role in the generation of ATP in invertebrates when a rapid energy supply is necessary [81,82,83].

One of the most characteristic features of the gill proteome, are the enzymes belonging to the amino acid and amino sugar pathways (spot no. 34, 42, 79, 103, 125 and 172), which are of great importance not only for the anabolism of the mussel but also for its osmotic integrity. Blue mussels are osmoconformers, which means that osmotic pressure and ionic composition of the haemolymph closely matches that of the salt or brackish water of their habitats. In addition to classical inorganic ions, such as sodium and chloride, highly soluble amino acids are used as intra-cellular osmotic buffer [84]. During hypertonic stress, the accumulation of intracellular alanine requires an inhibition of the pyruvate dehydrogenase complex (spot no. 113) in order to shunt mitochondrial pyruvate towards alanine and a high activity of the cytosolic malate dehydrogenase enzyme (spot no. 136) to maintain the cytosolic redox balance [85]. These metabolic processes are also involved in resistance to hypoxia during prolonged emersion [86]. The V-type proton ATPase catalytic subunit (spot no. 21) may also be related to ionic regulation via the gill epithelia, since V-type proton ATPase contributes to the buffering of the hypoxia-induced acidosis through the exchange of H^+^/Ca^2+^ during water deficiency [87]. Furthermore, low tide emersion usually signifies cessation of foraging; the animals usually pass this period fasting and in metabolic depression. Among proteins involved in homeostasis, Sirtuin 5 (spot no. 124) activates the mitochondrial carbamoyl-phosphate synthase through desuccinylation and thereby contributes to the regulation of blood ammonia levels during prolonged fasting. Sirtuins have been also shown to induce protein deacetylation, thus affecting the heat shock response in blue mussel congeners [14].

### 3.4. Antioxidant and Defence Systems

Gills constitute a privileged interface with the external medium and therefore gill epithelia comprise one of the first lines of defence against pathogens, xenobiotics and other environmental stressors. Consistently, several proteins belonging to the innate immune system have been identified such as spot no. 181 (C1q domain containing protein MgC1q64, putative) and no. 141 (cathepsin L, predicted). C1q domain containing proteins act through the recognition of pathogen associated molecular patterns (PAMPs) and possibly have an opsonin function [88,89]. The C1q domain is also present in heavy metal binding HIP (spot no. 110), which also has been detected in gills of clams [90]. Besides, haemocytes of *Ruditapes decussatus* subjected to bacterial challenge showed up-regulation of EST transcripts sharing similarities with this protein, highlighting a possible role in the immune defence [91]. On the other hand, the binding of divalent metal cations probably constitutes the major function of these proteins in gills, where they could contribute to metal detoxification processes.

Heavy metals, but also transition metals and organic compounds, which mussels are likely to encounter, notably in polluted habitats, are responsible for cellular oxidative stress through depletion in molecular thiol-containing antioxidants, catalysis of redox reactions and metabolism-induced bioactivation, respectively [92,93]. Life in the intertidal zone is also associated with hypoxia during emersion at low tide and reperfusion of oxygen in the initial reimmersion phase resulting in oxidative stress, which, in turn, will induce the antioxidant defence. For instance, the tyrosine 3-monooxygenase/tryptophan 5-monooxygenases, also named 14-3-3 epsilon proteins (spot no. 128, 129 and 130), play a central role in the regulation of signal transduction associated with the cellular redox status. During hypoxia they translocate into the nucleus and interact with the c-Jun N-terminal kinase (JNK) during oxidative stress [94]. Also, several enzymes that are involved in redox balance control were identified: catalase (spot no. 59); unspecified-Cu/Zn- and Mn-superoxide dismutases (SOD) (spot no. 175, 190 and 191); dyp-type and thioredoxine peroxidases: thioredoxin 1 (spot no. 203); thioredoxin peroxidase (spot no. 178); peroxiredoxin proteins (spot no. 170 and 184); sigma and beta glutathione transferases (GSTs) (spot no. 158 and 173). Catalase and SOD constitute the main antioxidant enzymes, which catalyse the reduction of reactive oxygenated species. Their activities are highly modulated in *Mytilus* spp. gills in response to different adverse environmental conditions [10,95,96,97]. Likewise, peroxiredoxins, peroxidases and, indirectly, thioredoxin participate in the reduction of H_2_O_2_ and other organic peroxides. Moreover, thioredoxin peroxidase could be involved in transcriptional induction of thioredoxin-system components in response to oxidative stress [98]. Thiol oxidoreduction reactions are crucial to cellular antioxidant processes and, together with glutathione metabolism, form a faculty, which is critical in a tissue subjected to frequent oxidative stress. The expression of two classes of GSTs (spot no. 158 and 173) in mussel gills is in agreement with this statement. GSTs, which are involved in the second phase of organic xenobiotic metabolisation (glutathione conjugation), can display peroxidase activity as well and exhibit particularly high levels of total activity in *M. edulis* gills [99,100].

### 3.5. Protein Stabilisation, Folding and Sequestration

Chaperones are multifunctional proteins, which assist protein folding and sorting [101,102,103], and are involved in various cellular processes such as growth, differentiation, and apoptosis. Heat shock proteins (Hsps) of the various families (small Hsp-, Hsp60-, Hsp70- and Hsp90-family) and their respective cognate forms (heat shock cognates, Hscs) belong to the most abundant cytosolic proteins. Their extensive presence in the gill proteome of *M. edulis* may be partly explained by the exigencies imposed to the gill tissue through the varying external physico-chemical conditions like salinity/osmolarity, temperature and/or desiccation. Stressors, such as oxidative stress and temperature changes, may induce cellular chaperones or heat shock proteins (Hsps) and elevated levels of these proteins help the animals to resist adverse environmental conditions by stabilising damaged proteins, which then may either be refolded or subjected to ubiquitin-mediated degradation by the proteasome (see next section). Thus, not surprisingly, Hsps and Hscs were particularly well represented in the mussel gill proteome (17% of the identified proteins). The classical protein extraction protocol using mechanical homogenisation, sonication and high molar urea releases a variety of stress proteins, notably of the Hsp70-family, which originate from different cellular compartments, such as the cytosol, nucleus, mitochondria or endoplasmatic reticulum (ER). This becomes particularly obvious in the 78 kDa and 94 kDa glucose regulated protein (spot no. 13 and 4), as well as other chaperones from the ER, like calreticulin (spot no. 68), endoplasmic reticulum protein ERp29 (spot no. 146) and the protein disulfide-isomerases (spot no. 55 and 70). Other Hsps are typically found in the mitochondria, e.g., Hsp60 (spot no. 39). The majority of the Hsps, however, probably represent cytosolic forms (spot no. 23 and 24: Hsp70; spot no. 20: Hsc71; spot no. 12: Hsp90; spot no. 166 and 168: small Hsp 22; spot no. 131–133, 144, 145 and 148: small Hsp 24.1).

The family of small Hsps (sHsps) comprises a suite of chaperones with variable Mr, ranging from about 15–30 kDa (average Mr *ca.* 17.9 kDa). They consist of monomeric or dimeric subunits that are composed of a conserved “α-crystallin” domain and variable *N*- and *C*-terminal regions [104]. This basic primary sHsp structure may be complemented with a “middle domain” or additional α-crystallin domains. The mono- or dimeric building blocks assemble into highly dynamic oligo- to multimeric polyhedrons (12mer–48mer) with molecular masses exceeding 200 kDa. The degree of oligomerisation and the exchange of subunits may depend on thermal or other environmental stresses [105]. sHsps often carry PTMs on the *N*-terminal region [104]; also here the phosphorylation status may determine chaperone activity and affect cellular distribution (reviewed in [105]). In addition, the subunits recovered from 2-DE gels may frequently be truncated of their terminal regions [104]. Hence, it is not surprising that identifications can be obtained for sHsp-proteoforms at various Mr and pI. Although their functional role is less studied than that of the Hsp70- or Hsp90-families [106], their response to thermal and other types of environmental, physiological and pathological stresses is well known [107]. Generally, they are considered as “holdases” that stabilise nascent or damaged proteins, thus preventing their aggregation [104] until the “foldases”, such as Hsp70 and Hsp90 assure (re-)folding of the destabilised proteins or direct them to degradation by the proteasome [45,108]. This function as holdase becomes particularly important whilst emersion during ebb occurs when the mussels’ depressed metabolism does not allow for excessive production of foldases, notably Hsp70. Furthermore, sHsps could have an important role in protecting proteins from oxidative stress that will inevitably occur when reimmersed during rising tide [49].

The Hsp70-family is by far the best-investigated and most eminent class of chaperones, being highly conserved across all domains of life. Several isoforms fulfil different cellular functions, with Hsc70 occupying a central role in chaperone-mediated protein folding [101]. Its inducible counterpart Hsp70 is one of the major stress-proteins and responds particularly to thermal stimuli but also to many other abiotic and biotic stressors [109,110]. Hsp70 appears to be a key-player, notably in fluctuating environments where its inducibility appears to be much higher than in more stable conditions [111,112,113]. Also, high numbers of Hsp70 genes were found in the oyster genome, probably reflecting the adaptation to harsh changes in the intertidal environment [2]. Interestingly, the gene structure of the promoter region of Hsps in organisms inhabiting fluctuating environments with regular exposure to abiotic stresses appears to be highly complex, as demonstrated by Pantzartzi *et al.* [12]. It is very likely that this complexity reflects the presence of various response elements that allow for a fine tuned and differential regulation of the numerous Hsps according to the respective stressor. For instance, specific regulation of Hsp70 isoforms in roots of *Musa* spp. through osmotic stress could be related to a specific abscisic acid response element present in the promoter region of some isoforms but not in others [114]. Hsp70s of *M. edulis* appeared in horizontally adjoining spots (of which spots no. 23 and 24 have been identified), being indicative of PTMs. *C*-terminal phosphorylation of Hsp70, which is supposed to regulate co-chaperone binding that changes Hsp70-function between folding and directing proteins for degradation [45], would be one possible explanation for this observation.

Hsps of the 90 kDa-family assist in ATP-dependant protein folding, whereby some Hsp90 closely interact with Hsc70, a cooperation coordinated by a number of co-chaperones that regulate Hsc70/Hsp90 activity through ATPase cycling and substrate exchange, thus forming a “multichaperone machinery” [101]. Hsp90 also mediates stress signal transduction via protein kinases and transcription factors through which stress inducible genes can be regulated [115,116]. In fact, proteotoxic stresses are less likely to change overall Hsp90 levels [117], but rather act through release of heat shock transcription factors that activate gene-expression via the heat shock response elements in the promoter regions of stress responsive genes [118]. Hsp90 may also regulate stress responses via MAP kinase signalling, which, for instance, may lead to cell wall modifications [118].

### 3.6. Intracellular Protein Trafficking

The t-complex protein 1 (TCP1), of which most of all subunits belonging to its functional ring could be identified (spot no. 33, 40, 43–45, 48 and 56) may be particularly representative of the intracellular transport of proteins. TCP1, also known as the TCP1 ring complex (TRiC), consists of two identical stacked rings, each containing eight different proteins. Although TCP1 belongs to the cytosolic compartment, where it assists the folding of proteins upon ATP hydrolysis, it may also be involved in the assembly of the BBSome, a complex participating in ciliogenesis, by regulating transport vesicles to the cilia. Organisation of the cilium as an extracytoplasmic organelle requires vesicular trafficking; a process modulated by small GTPases of the Rab- and Arf-families and which uses microtubule-dependent motor proteins to mobilize ciliary cargo [119]. Hence, TCP1 is likely to be an important component of cilia formation, an obviously eminent process in an organ that possesses a large amount of cilia such as gills.

The abundance of major vault protein (spot no. 6–9) most likely also relates to specificities of the gill structure. Briefly, vaults are multi-subunit structures that consist in huge cage structures of 12.9 mDa formed by dimers of half-vaults. Each half-vault comprises 39 identical major vault proteins of 110 kDa, PARP4 and one or several vault RNAs, small RNA species of 140 nucleotides that are involved in nucleo-cytoplasmic transport as well as in multiple cellular processes. Higher expression of vaults has been observed in epithelial cells with secretory and excretory functions, as well as in cells chronically exposed to xenobiotics, such as bronchial cells. In humans, the phosphorylated protein interacts with the SH2 domains of proteins, modulating their effects [43].

### 3.7. Ubiquitin Proteasome System

The Ubiquitin Proteasome System (UPS; [120]) was well represented in the *M. edulis* gill proteome, with a total of 12 spots identified. Although detailed information concerning the role of UPS in the gills of bivalves remains scarce [121], several proteosomal components have been repeatedly detected in earlier “omics” studies on bivalves [14,122,123]. The UPS is a highly conserved system responsible for cell clearance of abnormal, damaged proteins or those that are no longer of physiological relevance in the cell. Thus, the UPS constitutes the main cellular system implied in controlled protein degradation. Briefly, proteins targeted for degradation are first labelled with polyubiquitin tags through a three-step cascade, and then recognized, unfold and finally cleaved into short peptides by the 26S proteasome. 

Ubiquitination requires the sequential action of three types of enzymes: ubiquitin is first activated by E1, then transferred to E2 ubiquitin-conjugating enzyme and finally, an E3 ubiquitin-ligase attaches the ubiquitin moiety to the substrate. Spot 17 corresponds to Tripartite Motif containing protein 33 (TRIM33) also known as TIF1γ, a nuclear RING-based E3 ligase. It is implicated in regulation of TGF-β pathway through promoting ubiquitination of smad4 [124]. More recently, Kulkarni *et al.* [125] demonstrated that TRIM33 is involved in double strand break response.

The 26S proteasome consists of a 20S catalytic core particle linked to one or two 19S regulator complexes containing regulatory proteins (RP) [126,127]. The proteolytic core is a barrel-shaped complex composed of two external rings of seven α-subunits (α1–α7) that embrace two inner rings of seven β-subunits (β1–β7). The α-rings regulate the entry into the catalytic chamber through their conserved *N*-terminal extensions [128]. We identified five spots corresponding to the 20S proteasome: spots no. 169, 147, 142 and 153 were identified as subunits α2, α4, α5 and α6, respectively, and spot no. 177 corresponded to a non-active β-subunit, namely β6, implied in the maturation of the three active β-subunits, which carry proteolytic activities [129,130]. Alternative forms of the proteasome have been described for jawed vertebrates (*i.e.*, the immunoproteasome, [126]), in which variants of three of the β-subunits replace the classical ones of the 20S core. Apparently, such β-subunits are restricted to vertebrates, as none of these alternative subunits was identified in our gill proteome.

The 19S RP can be dissociated into a lid and a base covered by the lid. We identified a single subunit from the lid: RPN11 (spot no. 120), which is a deubiquitinating enzyme (DUB) and belongs to the metalloenzyme JAMM-family. It appears to promote substrate degradation through cutting at the base of the polyubiquitin chain [131]. Recently, it has been suggested that RPN11 could be implicated in response to double stand breaks in mammals [132]. The base of RPN11 consists of six ATPase and four non-ATPase subunits and is involved in recognition, unfolding and translocation of protein into the core particle. Spots no. 69 and 96 correspond to the ATPase subunits RTP3 and RTP4, respectively, and spots no. 76 and 77 were identified as RTP5, an ATPase subunit specifically implied in the recognition of the polyubiquitylated substrate [133].

## 4. Conclusions

The data presented in this study extend our knowledge of the *M. edulis* gill proteome. Despite a weak representation of this species in gene and protein databases, we were able to identify more than 100 proteins and more than 200 proteoforms present in the mussel gill tissue. Although many of the identified proteins are of ubiquitous nature, which also explains their abundance, many of the functional groups to which they could be attributed display plausible relations to the general stress response, the distinctive structural features of the gill tissue and the metabolic demands of a highly dynamic environment: the main characteristics of gill organization and physiology are indeed underscored by an important representation of cytoskeleton, metabolism and defence related proteins, thus validating the protein identifications. The equivalence of this proteome inventory to those described by Tomanek and Zuzow [14] and Fields *et al.* [36] validates the importance of many of these proteins for a life in harsh environmental conditions. Identification and knowledge about the proteoforms being the first step, quantitative proteomics, investigating condition-related alterations of the proteome, will benefit from a thorough and comprehensive mapping of the proteome constituents (see for instance [134,135]) and, particularly, from the knowledge on protein species from the same protein [54]. In this respect, also the reproducibility of proteoforms, *i.e.*, the dispersion of their relative abundance among replicate gels, is important information. Several implications arise from the inventory presented here: firstly, a large number of stress-related proteins can be identified and localised simultaneously with some experience on 2-DE gels, allowing for characterisation of complex protein networks and their perturbations. Proteomics, potentially, enables a more comprehensive view on particular response-complexes such as oxidative stress- and Hsp-networks or the proteasome [136]. Alterations of specific protagonists within these complexes may deliver more detailed information about the underlying molecular mechanisms, and quantitative changes, rather than focussing on one particular marker protein or conducting several independent assays. Secondly, measures of total protein, for instance using an immunoassay, may indicate elevated protein levels which, however, may comprise an ill-defined amount of non-functional protein (e.g., truncated forms or PTMs that inhibit protein activity). Indeed, McDonagh and Sheehan [137] demonstrated increased carbonylation and ubiquination of proteins in response to oxidative stress, pointing to irreversible protein damage [137]. But oxidative stress may also change the redox status of proteins, with protein oxidation representing an important regulatory modification [138]. Proteins for which deviations from their expected PI and Mr were also detected in this study, such as β-tubulin (spot 61), calreticulin (spot 68), protein disulphide-isomerase (spot 68), enolase (spot 74), gelsolin (spot 78) and heavy metal-binding protein (spot 110) were found to be oxidised by the model pro-oxidant menadione, leading to the reduction of free thiols and an increase of disulphides [138]. Thus, close examination of the different proteoforms displayed on 2-DE gels and their quantitative changes could reveal the precise nature of protein accumulation and modification following changes of the environmental conditions or exposure to toxic compounds, thus providing an in depth examination of the stress responses. Indeed, one of the strengths of the gel-based proteomics approach is the potential for analysing various PTMs associated with different states of the animal and its surrounding environment. Albeit being a complicated endeavour, examination of putative PTMs should be given more weight as this could provide supplementary and more far reaching information for the interpretation of the complexity of stress responses, which help these animals to cope with their ever changing environment and to fight parasite infestation or exposure to man-made chemicals. Our increasingly comprehensive catalogue of mussel gill proteins represents a valuable resource for future studies of responses to environmental and anthropogenic stresses in *Mytilus* spp.
